# Dichotic Listening Test (directed attention mode) in children with cleft lip and palate

**DOI:** 10.1016/S1808-8694(15)31374-4

**Published:** 2015-10-17

**Authors:** Isabel Cristina Cavalcanti Lemos, Camila Zotelli Monteiro, Renata Arruda Camargo, Ariane Cristina Sampaio Rissato, Mariza Ribeiro Feniman

**Affiliations:** 1 MSc, Speech and Hearing Therapist; 2 Speech and Hearing Therapist, scholarship holder; 3 MSc, PhD student; 4 Speech and Hearing Therapist, MSc student; 5 PhD, Associate Professor. Faculdade de Odontologia de Bauru - Universidade de São Paulo

**Keywords:** attention, hearing, cleft palate

## Abstract

Conductive hearing loss in the first years of life may lead to hearing processing and attention deficit disorders, and consequently to communication and learning impairments.

**Aim:**

this paper aims to examine the performance of children with cleft lip and palate in dichotic listening tests (directed attention mode) and compare them to a control group without cleft lip and palate.

**Materials and method:**

fifty-two children of both genders were enrolled in the study. Their ages ranged between 7 years and 7 years and 11 months, and they were divided into two groups: a study group featuring children with cleft lip and palate (n=27) and a control group with children without this anomaly (n=25). The children were first interviewed, then underwent a series of conventional hearing tests, and lastly were applied the dichotic hearing test.

**Results:**

when submitted to the dichotic listening test (directed attention mode), the children in the study group had lower scores for both ears when compared to those in the control group. Statistical significance was found for variable gender in the groups, with p=0.026.

**Conclusion:**

in the dichotic listening test only the girls with cleft lip and palate had lower scores than the girls in the control group. This is a prospective clinical study.

## INTRODUCTION

Congenital cleft lip and palate (CLP) develops during embryo and early fetal life and is clinically manifested by the absence of closure in the lip, palate, or both. This population is almost universally affected by otitis media with effusion (OME) associated with Eustachian tube involvement. Studies done with children suffering from CLP found that 40% of them had normal peak pressures under tympanometry^1^ and 25% had conductive hearing loss ranging from mild to severe^2^.

OME is frequently found in this population as the palatine muscles have not fused, thus reinforcing the idea that middle-ear hypoventilation may induce the onset of otitis media with effusion. As the palatine tensor and elevator muscles do not have support on the contralateral side, they cannot effectively open the Eustachian tube due to the deformities present in the cartilaginous framework^3^.

OME is a special, silent type of otitis media, characterized by the accumulation of a serous, mucous, glue-like fluid in the middle ear. This disease is currently one of the most common causes of hypoacusis - often bilateral - in children of up to 10 years of age^4^.

Although hearing acuity is supposed to be present in the fetus, that does not mean the child understands what he or she hears to thus use it as a tool for communication. In order for that to happen, he or she must acquire the skills to enable the interpretation and analysis of the sounds detected by the peripheral auditory system^5^.

Children frequently diagnosed with hypoacusis due to OME may experience impairments in the development of their listening skills, once a peripherally altered auditory system may not be able to properly decode the messages directed to the child, leading the listener to receive distorted, incomplete messages. The development of the listening skills associated with auditory processing relies upon the innate biological capabilities of the individual, as well as the experiences he or she has had. Changes in those skills may lead to lower school performance, delayed linguistic development, difficulty understanding what others say, and learning challenges.

Studies looking at auditory processing have observed that 62% of the children with history of recurrent otitis media have alterations in at least one listening skill. The most compromised skills are figure and ground, binaural integration, and sequential memory. It is therefore suggested that fluctuating hearing occasioned by recurrent OM may negatively impact normal development, as impaired listening strategies might persist even when the disease is inactive^6^.

It is possible to shed light on the competencies, skills, and capacities in dealing with sound by observing the responses children and adults of various age ranges produce when performing a wide range of tasks^7^, such as those under dichotic listening tests, in which two different sounds are presented to each ear at the same time. The dichotic listening test (directed attention mode) is used to assess the figure and ground skill for verbal sounds during sustained attention7 and selective attention processes^8^.

Santos^9^ (1998) studied the performance of 140 right-handed individuals with ages varying between 5 and 25 years under the dichotic listening test, both for binaural integration and directed attention to the right and left. Digit recognition was above 90%, showing that the dichotic listening test is easy to carry out and can be applied to individuals as young as five years old. The author concluded that the participants had right-ear advantage on both stages and that test performance improved as age increased.

Conductive hearing loss in the first years of life may lead to auditory processing and attention disorders, and consequently learning and communication impairments.

Therefore, this paper aims to examine the performance of children with cleft lip and palate in dichotic listening tests (directed attention mode) and compare them to a control group without cleft lip and palate.

## MATERIALS AND METHOD

### Materials

Fifty-two children of both genders with ages ranging from 7 years to 7 years and 11 months were enrolled in this study. They were divided into two groups:

• G1: control group formed by children without CLP
• G2: study group formed by children with CLP

Enrollment criteria for G1

1) Absence of cleft lip and palate and diagnosed syndrome
2) Absence of diagnosis for attention deficit and hyperactivity disorder (ADHD) and/or taking medication to treat ADHD
3) Having peripheral hearing within normal ranges
4) Being right-handed
5) Not presenting complaints indicative of altered auditory processing

Enrollment criteria for G2

1) Having transforamen or postforamen cleft lip and palate10
2) Absence of diagnosis for attention deficit and hyperactivity disorder (ADHD) and/or taking medication to treat ADHD
3) Having peripheral hearing within normal ranges
4) Being right-handed
5) Absence of diagnosis for any sort of syndrome

### Selection Procedure

G1 participants were gathered from two public schools located in the city of Bauru, São Paulo. A letter (Annex 1) was sent to the parents of all students enrolled in the first grade (190 students), explaining the relevance of this study and asking those interested in enrolling their children to fill out a form with the child’s name, name of the parent or caretaker, and contact phone number. Next, the researcher called the parents who responded to the letter and scheduled meetings with them and the child. The parents of seventy children responded to the letter, and all of them were called over the phone. Fifteen could not be reached either because the given phone number was incorrect, the line was busy, or phone service had been terminated. The children who failed to come to the first scheduled visit were given a second chance and were called again. Those who failed to show up for the second time were excluded. Eleven children were excluded for not showing up the second time. The other 44 showed up; 25 of them were enrolled on G1; the remaining 19 failed to meet the enrollment criteria.

G2 patients were screened from a list produced at the Data Processing Center containing all cleft lip and palate and cleft palate only patients that had been seen between January and September of 2006 and were seven years old at the time they were seen. The scheduling service was then asked to see if it would be possible to fit the study tests into the pre-existing appointments. It was thus possible to schedule 150 visits. Thirty-three did not show up, 90 were excluded for not meeting the enrollment criteria for G2, and 27 cleft lip and palate or cleft palate children were included in G2, five with postforamen and 22 with transforamen cleft lip and palate.

Table 1 shows the distribution of the sample between the two groups, according to gender.

**Table 1:** Number of individuals on each group by gender.

Gender	Group 1	Group 2	Total
Female	13	13	26
Male	12	17	29
Total	25	30	55

## METHOD

This study was approved by the Research Ethics Committee of our institution under permit 233/2005.

For both groups, the evaluation process was based on a questionnaire that aimed at obtaining the information to assign the children to either of the groups and to check for their auditory health and aspects related to the children’s attention status; a set of conventional hearing tests; and the dichotic listening test (directed attention mode)11. All the procedures were carried out on one same day, in the order they were described.

The conventional hearing tests performed are:

• Tone threshold audiometry: hearing thresholds were tested for 250 Hz, 500 Hz, 1 kHz, 2 kHz 3 kHz, 4 kHz, 6 kHz, and 8 kHz.
• Impedance test: tympanometric curves were obtained with the 226 Hz probe and ipsilateral and contralateral acoustic reflexes captured for 500 Hz, 1 kHz, 2 kHz, and 4 kHz.

This set of tests was carried out before the dichotic listening test to exclude individuals with peripheral hearing loss and altered middle ear function.

Parents and caretakers signed a free informed consent document after reading the informative letter.

The dichotic listening test (directed attention mode) consists of four presentations of a list of two-syllable messages in Brazilian Portuguese, in which four different messages are presented simultaneously, two on each ear, thus characterizing a dichotic task. The list designed by Santos; Pereira^11^ (1997) contains 40 randomly arranged message pairs, adding up to 20 presentations with two pairs each, a pair for each ear. The messages used to form the list are the numbers four, five, eight and nine. The first time they are played, the patient is told to repeat only those he or she heard on the right ear (directed attention to the right). The second time they are played, the focus lies on the left ear (directed attention to the left). Then the ear phones are switched to the opposite ears and the test is repeated for both ears. A two-channel audiometer (SD 50) connected to a CD player was used in the tests at 50 dBNS.

All results were recorded in a score sheet containing the four lists that make up the test. Accurately identified messages were circled. Then the number of accurately identified messages for right and left ears was added up.

Variance Analysis (ANOVA) was used to statistically treat the data, with repeated measurements for three factors to verify the associations between the dichotic listening test and variables ear, gender, and group, being ear the repeated factor. Statistically significant differences were observed when p≤0.05.

## RESULTS

Dichotic listening tests (directed attention mode) were performed with the 52 children that participated in the study.

The results for right and left ear for both groups are shown on Table 2.

**Table 2:** Average, standard deviation, minimum, median, and maximum values for right and left ear in the dichotic listening test.

Variable Response	Group	N	Average	SD	Minimum	Median	Maximum
Right Ear	1	22	90,85	7,76	75,00	90,00	100,00
	2	30	82,75	12,57	46,25	86,88	98,75
Left Ear	1	22	87,90	7,04	75,00	87,50	97,50
	2	30,00	79,96	11,86	52,50	78,75	98,75

Table 3 shows the percentage averages of right answers in the dichotic listening test for each ear and group.

**Table 3:** Percentage of right answers under the dichotic listening test for right and left ear of both groups

	Right Ear	Left Ear
Group 1	90,85%	87,9%
Group 2	82,75%	79,96%

The variance analysis model with repeated measurements for three factors (group, gender, and ear), ear being the repetition factor, was used to look for associations between the variables and the dichotic test results. Table 4 shows that the relationship between Group and Gender is statistically significant (p=0.026).

**Table 4:** Variance analysis with repeated measurements for the dichotic listening test.

Factors	p
Group	0,001*
Gender	0,160
Ear	0,078
Gender* Group	0,026*
Group* Ear	0,891
Gender* Ear	0,627
Gender* Group* Ear	0,593

The Bonferroni^12^ correction was used to allow for post hoc comparisons. The results gathered on Table 5 indicate that the control group presented statistically higher scores than the CLP group (p<0.0001); it was also found that the boys had a statistically significant higher score than the girls in the group with cleft lip and palate (p=0.0059).

**Table 5:** Post hoc comparison of variance analysis with repeated measurements.

Difference	Estimate	Standard error	gl	t	p	*ns *	Confidence interval (95%)**	
							Lower Limit	Upper Limit
g1-g2 M	3,2	3,3	48	0,96	0,3431	0,0125	-5,4	11,7
g1-g2 F	13,7	3,1	48	4,35	<0,0001*	0,0125	5,5	21,8
M-F g1	-2,0	3,5	48	-0,58	0,5674	0,0125	-11,0	7,0
M-F g2	8,5	2,9	48	2,88	0,0059*	0,0125	0,8	16,1

* Significance level adopted considering the Bonferroni correction

** Confidence interval adjusted according to the Bonferroni correctioni

## DISCUSSION

During the dichotic listening test (directed attention mode), we observed that the CLP group had lower scores than the control group for both ears (Table 3).

Through the dichotic listening test (directed attention mode), we could assess the mental skills of figure and ground and binaural separation.

Mental skills can be analyzed when specific cognitive function maturity and the corresponding neural development are correlated, and thus the role experience has in shaping the mind and the brain^13^. Experience plays a critical role in the final growth and accurate synchronization of the cerebral neural circuits^14^. During the development of the nervous system, some periods are critical for the achievement of normal outcomes, such as when neurons compete for synaptic sites; this way, the nervous system optimizes the neural connections during this period^15^. Therefore, the development of listening skills depends on the perception of auditory stimuli.

Modifications in the amplitude of tympanic stimuli change the rate at which neurons are triggered^13^, thus showing a close relationship between auditory stimulus perception and development of listening skills. We may therefore infer that sensorial deprivation secondary to middle ear infection may impair the development of listening skills.

Statistically significant interactions were found for gender on both groups, with a p=0.026 (Tabela 4), which led us to look separately at both genders in the groups. The girls in the control group had significantly higher scores than the girls in the CLP group (p<0.0001); boys, however, had no statistically significant difference between groups. In the group with cleft lip and palate, the boys performed better than the girls (p=0.0059), while in the control group such difference was not observed (Table 5).

These results show that girls with cleft lip and palate had lower scores in the dichotic listening test. The literature does not mention such difference between genders in terms of right answers in dichotic listening tests using 1, 2, 3, or 4 pairs^16^, in non-verbal and consonant-vowel dichotic tests8. Santos9 (1998), using the same dichotic listening test applied in this study, was not able to find differences between genders in his groups of children.

Contrary to our findings, Jäncke; Steinmetz; Volkmann^17^ (1992) found that females had higher scores than males at the dichotic test using consonants and vowels. Hertrich et al.^18^ (2002) noted gender-related differences at the consonant-vowel dichotic test for artificial stimuli. When the stimulus was natural speech, such difference was not observed.

One should be very careful when comparing different dichotic tests, once different dichotic tasks produce different outcomes^17^.

There seems to be a trend of male individuals presenting greater right-ear advantage than females^18^, ^19^. However, Bellis; Wilber20 (2001) in their study using dichotic listening tests in adults, and Ortiz8 (1995) using the consonant-vowel and non-verbal tests, did not observe that same trend. Likewise, this study showed that ear was not a factor with statistically significant difference, p=0.078 (Table 4).

In order for one to perform well under a linguistic dichotic task, the information needs to go through the corpus callosum before it reaches the dominant hemisphere for language^21^, ^22^. Morton; Rafto^23^ (2006) found that the greater the number of nerve fibers in the corpus callosum, the better is the performance at dichotic tasks with consonant-vowel stimuli, being gender not a statistically significant factor.

In this study the difference found between genders must be carefully considered, once it is not in alignment with the literature and may be an isolated finding. In this case, further studies must be conducted so as to determine the influence of gender in dichotic listening test scores.

## CONCLUSION

The results observed in this study indicate that the performance of children with cleft lip and palate (directed attention mode) was different for boys and girls, as girls had lower scores than the ones in the control group. We must stress that one processing test is not conclusive to characterize whether a group is deficient, and the studied group may even be considered deficient to study the actual effectiveness of the test.
